# Changes in the Treatment for Out-of-Hospital Cardiac Arrest During the Initial Stage of the COVID-19 Outbreak in Japan

**DOI:** 10.7759/cureus.25502

**Published:** 2022-05-30

**Authors:** Kenji Numata, Chinami Sakurai, Michiko Mizobe, Yosuke Homma, Jin Takahashi, Hiraku Funakoshi

**Affiliations:** 1 Emergency Department, St. Marianna University Hospital, Kawasaki, JPN; 2 Department of Emergency and Critical Care Medicine, Tokyobay Urayasu Ichikawa Medical Center, Urayasu, JPN

**Keywords:** treatment algorithm, personal protective equipment, emergency department cpr, ohca, covid 19

## Abstract

Introduction: Cardiopulmonary resuscitation (CPR) for out-of-hospital cardiac arrest (OHCA) patients during the coronavirus disease 2019 (COVID-19) pandemic carries an added risk of COVID-19 infection for healthcare workers. However, because of the shortage of medical supplies and limited evidence of COVID-19 in the initial stages of the pandemic, strategies for the management of OHCA patients may have varied across hospitals.

Method: A web-based questionnaire was used. The first section collected data about physician characteristics. In the second section, participants responded "Yes" or "No," if they had made changes in the areas of "personal protective equipment (PPE)" or "CPR Algorithm" for OHCA patients (these changes were the personal views of the surveyed respondents). The questionnaire was sent to the members of the Emergency Medicine Alliance mailing list. The response period was from May 22 to June 5, 2020 (the first state of emergency related to COVID-19 was declared on April 7, 2020, in Japan). Participants were asked to indicate their stress level resulting from these changes using the Likert scale ranging from 1 to 10, where 1 = "no stress" and 10 = "severe stress."

Result: A total of 110 physicians responded during the study period. The majority of participants reported changes in "PPE" (n = 106, 96.4%) and "CPR Algorithm" (n = 86, 78.2%). The reported stress level due to changes in PPE was 8 (IQR 6-9) and due to changes in the CPR algorithm, it was 7 (IQR 5-8).

Conclusion: Findings of this study suggest that physicians experienced changes in care for OHCA patients and felt stress during the initial stage of the COVID-19 pandemic. Thus, it would be better to list the actual measures that can be undertaken to prepare for any future pandemics.

## Introduction

In late 2019, infection with a novel beta coronavirus, subsequently named the severe acute respiratory syndrome coronavirus 2, was reported in individuals who had visited a wet market in Wuhan, China. Since then, the virus has spread rapidly, which has led to the coronavirus disease 2019 (COVID-19) pandemic [1]. The first case in Japan was diagnosed on January 28, 2020. COVID-19 was qualified as a global pandemic by the WHO on March 11, 2020.

Cardiopulmonary resuscitation (CPR) for out-of-hospital cardiac arrest (OHCA) patients during the COVID-19 pandemic carries an added risk of COVID-19 infection for healthcare workers [2]. Physicians were recommended to wear personal protective equipment (PPE) to prevent the risk of COVID-19 infection during Advanced Life Support [3]. However, because of the shortage of medical supplies and limited evidence of COVID-19 in the initial stages of the pandemic, strategies for the management of OHCA patients may have varied across hospitals. This study aimed to investigate and clarify changes in the treatment of OHCA patients during the initial stage of the COVID-19 pandemic in Japan.

## Materials and methods

This was a web-based questionnaire study. The response period was from May 22 to June 5, 2020 (the first state of emergency related to COVID-19 was declared on April 7, 2020, in Japan). This study was approved by the Ethics Committee of the Tokyo Bay Urayasu/Ichikawa Hospital (approval number: 540).

Participants

The questionnaire was sent to the members of the Emergency Medicine Alliance (https://www.emalliance.org/) mailing list (a total of 3,233 physicians who engage in emergency room care were registered as of May 23, 2020). We included those who responded to the questionnaire.

Questionnaire

A web-based questionnaire was used (Appendices). The questionnaire consisted of two sections and 23 questions. The first section collected data about physician characteristics. In the second section, participants responded "Yes" or "No," if they had made changes in the areas of "PPE" or "CPR Algorithm" for OHCA patients (these changes were the personal views of the surveyed respondents). If they answered "Yes," details about these changes were asked. Participants were asked to indicate their stress level resulting from these changes using the Likert scale, ranging from 1 to 10, where 1 = "no stress" and 10 = "severe stress." We included physicians who answered the questionnaire. Values were given as percentages and median (interquartile range [IQR]).

## Results

A total of 110 physicians responded during the study period (Table 1). Of these, there were 90 males (81.1%), and the median age at post-graduation was 12 years (interquartile range (IQR): 7-19 years). Regarding specialization, 86 participants were emergency physicians (78.2%), 16 were internists (14.5%), and 3 were intensivists (2.7%). The number of beds at the hospitals where the participants worked was most commonly ≥500 beds (reported by 55 participants [50.0%]). The most common month in which COVID-19 was first noticed in real practice was February 2020 (reported by 49 participants [44.5%]).

**Table 1:** Characteristics of participant

Participant characteristics (n = 110)	
Postgraduate year, year, median (IQR)	12 (7–19)
Male sex, n (%)	90 (81.8%)
Specialty, n (%)	
Emergency physicians	86 (78.2%)
Internists	16 (14.5%)
Intensivists	3 (2.7%)
Other specialties	5 (4.5%)
Number of beds at the hospital where they work, n (%)	
20–99 beds	5 (4.5%)
100–199 beds	6 (5.5%)
200–299 beds	3 (2.7%)
300–499 beds	41 (37.3%)
≥500 beds	55 (50.0%)
The month in which COVID-19 was first noticed in practice, n (%)	
December, 2019	1 (1.0%)
January, 2020	12 (10.9%)
February, 2020	49 (44.5%)
March, 2020	34 (30.9%)
April, 2020	12 (10.9%)
May, 2020	2 (1.8%)

Table 2 shows the results of a questionnaire regarding the treatment of out-of-hospital cardiac arrest during the COVID-19 outbreak. The majority of participants reported changes in "PPE" (n = 106, 96.4%) and "CPR Algorithm" (n = 86, 78.2%). The most frequent response to the change in PPE was "full PPE with N95 filtering facepiece respirator" (n = 77, 77.0%), and the most common change to the CPR algorithm was "early intubation" (n = 33, 30%). The reported stress level due to changes in PPE was 8 (IQR 6-9) and due to changes in the CPR algorithm, it was 7 (IQR 5-8).

**Table 2:** Results of a questionnaire regarding the treatment of out-of-hospital cardiac arrest during the COVID-19 outbreak

Questionnaire about PPE	
Changes in PPE, Yes, n (%), (n = 110)	106 (96.4%)
Details of PPE changes, n (%), (n = 106)	
^*^Full PPE (with N95 respirator)	75 (70.8%)
Full PPE (with surgical mask)	20 (18.9%)
Gloves with N95 respirator	4 (3.8%)
Gloves with a surgical mask	5 (4.7%)
Depends on the situation	2 (1.9%)
Stress from changes in PPE, median (IQR), (n = 106)	8 (6–9)
Questionnaire about CPR algorithm	
Changes in CPR algorithm, yes, n (%), (n = 110)	86 (78.2%)
Details of CPR algorithm changes (participants can select multiple answers), n (%), (n = 78)	
Early intubation	33 (42.3%)
Early termination of resuscitation	12 (15.4%)
Reduced number of resuscitation team members	10 (12.8%)
Interruption in chest compression during intubation	9 (11.5%)
Changes in resuscitation room (isolation room)	9 (11.5%)
Use of intubation box	5 (6.4%)
Intubation only performed by an expert	4 (5.1%)
Hands-only CPR	2 (2.6%)
Stress from changes in CPR algorithm, median (IQR), (n = 86)	7 (5–9)

## Discussion

Most physicians reported changes in the PPE and CPR algorithms after the COVID-19 pandemic. However, the details of PPE changes and CPR algorithm changes were different among physicians. These differences might have been caused by a shortage of resources and a lack of evidence regarding best practices for COVID-19 at that time [4,5]. In the United States, perhaps the earliest example was the near-immediate realization that there were insufficient high-filtration N-95 masks for healthcare workers, prompting contingency guidance on how to reuse masks designed for single use [6]. A shortage of personal equipment has also been reported in Japan [7]. We considered that these factors might affect the results.

The level of reported stress caused by both the changes was high, despite the difference in changes in PPE and CPR algorithms. It is known that such changes lead to stress, especially when the consequences of the changes are uncertain [8]. Moreover, during the COVID-19 pandemic, physicians had to make several unusual decisions, such as reducing the duration of CPR, as aerosols generated during CPR procedures for patients with COVID-19 could infect medical professionals. Hence, the CPR status must be determined early so that patient prognosis, provider safety, and PPE can be considered. Consequently, the earlier "do it all" approach for resuscitation probably no longer applies to OHCA patients [9]. Furthermore, although efforts have been initiated to contain the number of cases and extraordinary measures have been put in place, the dramatic increase in ICU admission of patients with COVID-19 abruptly overwhelmed the ICU capacity in Italy. Physicians have proposed directing crucial resources, such as intensive care beds and ventilators, to patients with COVID-19 who can benefit most from treatment during the pandemic [10]. These factors may have caused uncertainty and induced severe stress.

Sutherland and Cooper conducted a cross-sectional survey to compare general physicians’ stress and job satisfaction before and after the introduction of the new contracts [11]. General practitioners are increasingly required to work and cooperate within multidisciplinary teams with other independent professionals after starting the contraction. The survey revealed that general practitioners reported more stress, anxiety, and depression following the commencement of the contract (the highest mean change in stress was 0.85; stress was rated on a scale of 1 to 5). In our study, participants reported high stress change. It has been reported that physicians often fail to recognize symptoms of burnout or depression; moreover, they seek help less often [12]. It is important that the impact of change is carefully monitored to avoid any adverse impact (like depression or burn out).

This study had several limitations. First, the sample size was small, and the response rate was low, which can be attributed to the fact that the respondents were volunteers. The second concerns the external validity because we chose Emergency Medicine Alliance mailing list members to serve as participants; therefore, the risk of selection bias should be considered.

## Conclusions

Findings of this study suggest that physicians experienced changes in care for OHCA patients and felt stress during the initial stage of the COVID-19 pandemic. This stress might be caused by uncertainty. It is said that burnout is a state of emotional, physical, and mental exhaustion caused by excessive and prolonged stress. Thus, it would be better to list feasible measures (e.g., stocking of medical supplies and establishing contingency plans for healthcare workers) that can be undertaken and propose solutions to increase work satisfaction among physicians, to prepare for any future pandemic.

## Figures and Tables

**Figure 1:**
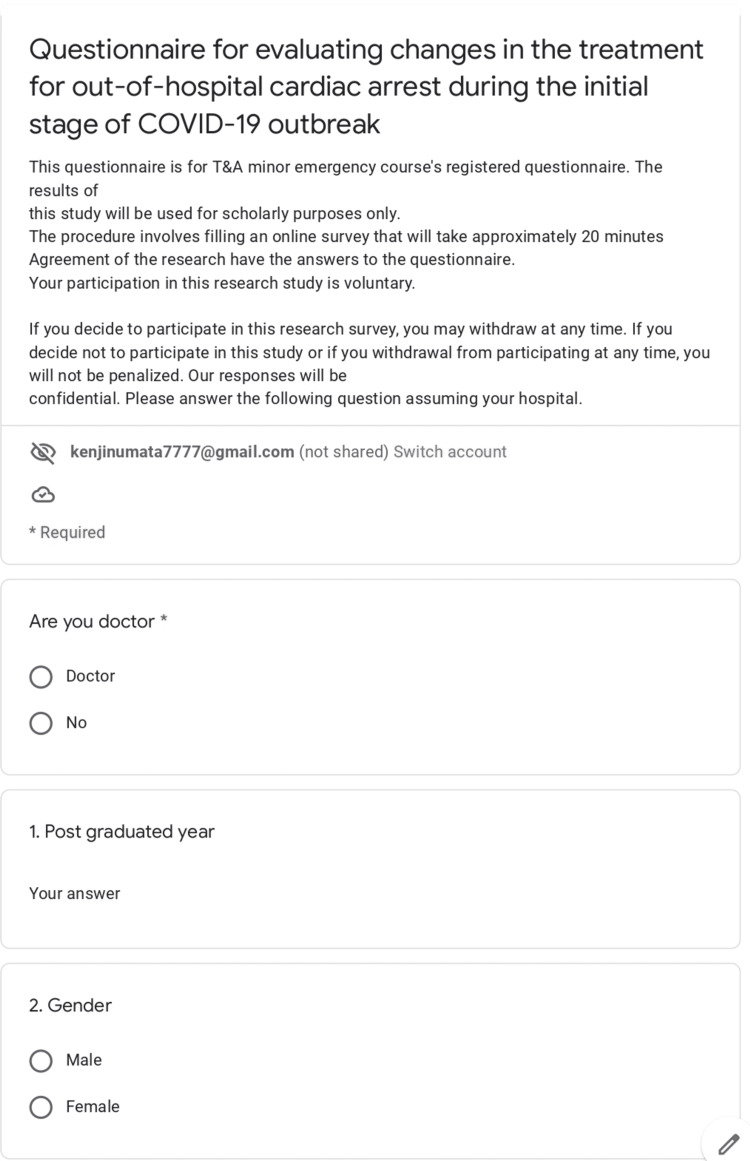
English questionnaire for evaluating changes in the treatment for out-of-hospital cardiac arrest during the initial stage of COVID-19 outbreak, Page 1

**Figure 2:**
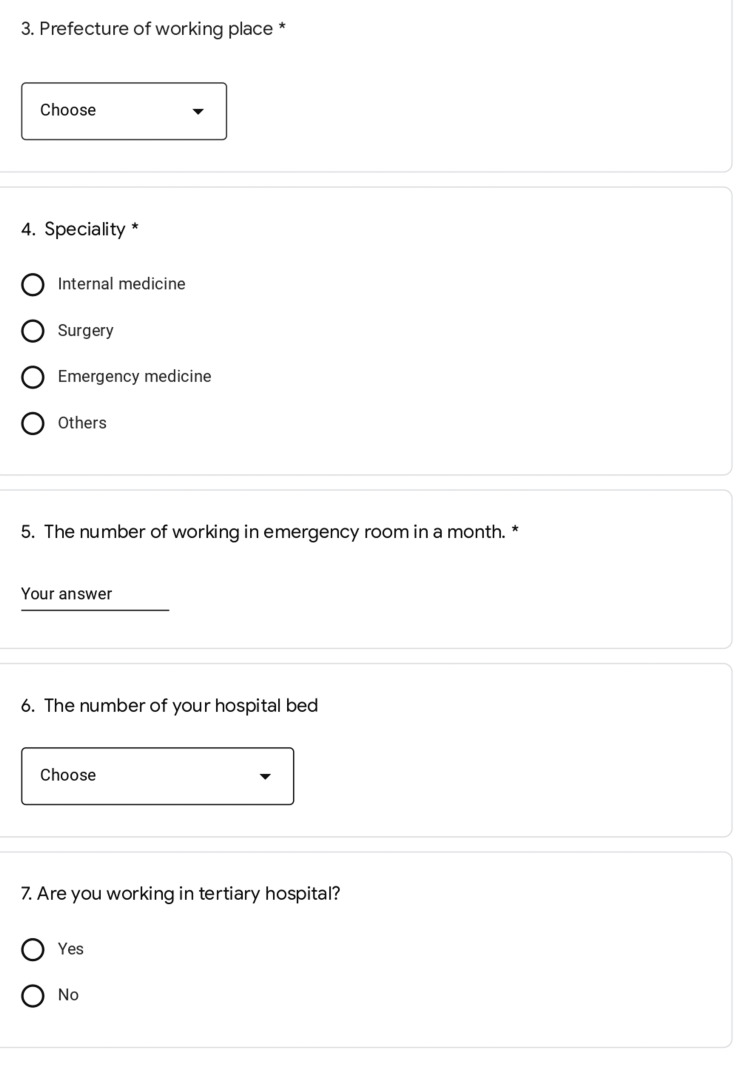
English questionnaire for evaluating changes in the treatment for out-of-hospital cardiac arrest during the initial stage of COVID-19 outbreak, Page 2

**Figure 3:**
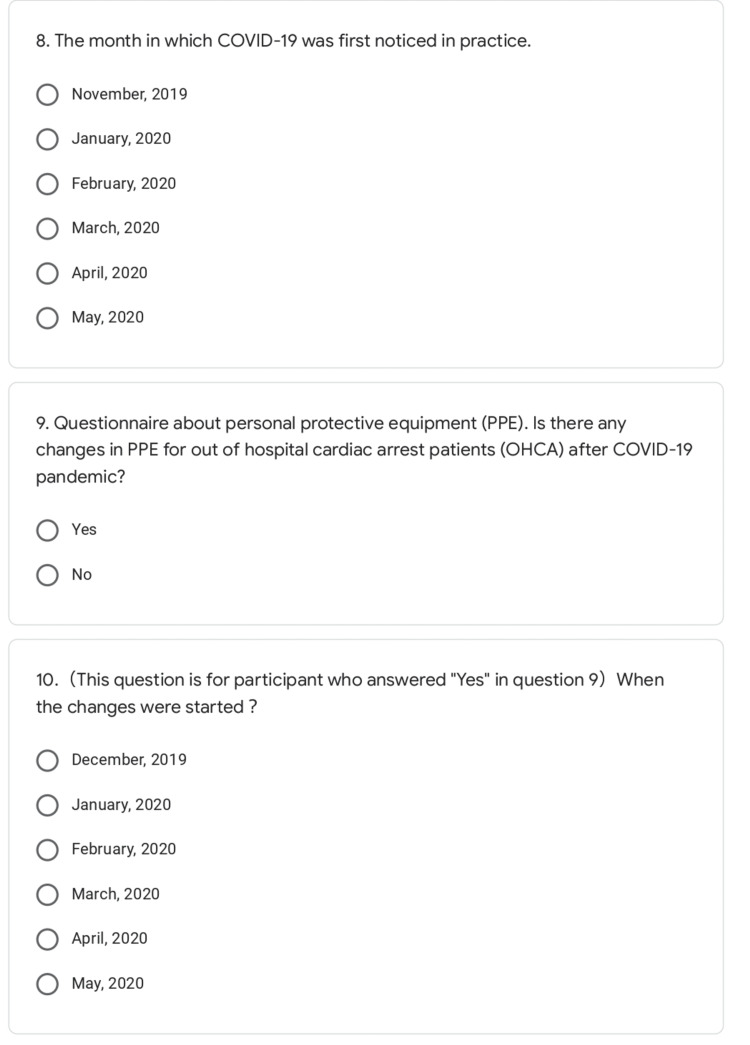
English questionnaire for evaluating changes in the treatment for out-of-hospital cardiac arrest during the initial stage of COVID-19 outbreak, Page 3

**Figure 4:**
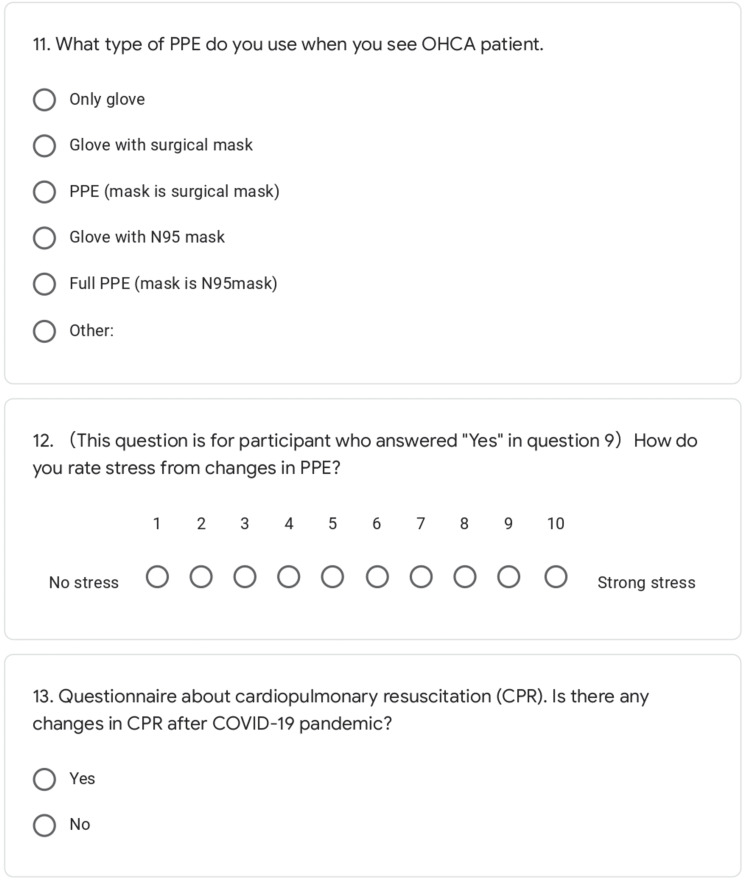
English questionnaire for evaluating changes in the treatment for out-of-hospital cardiac arrest during the initial stage of COVID-19 outbreak, Page 4

**Figure 5:**
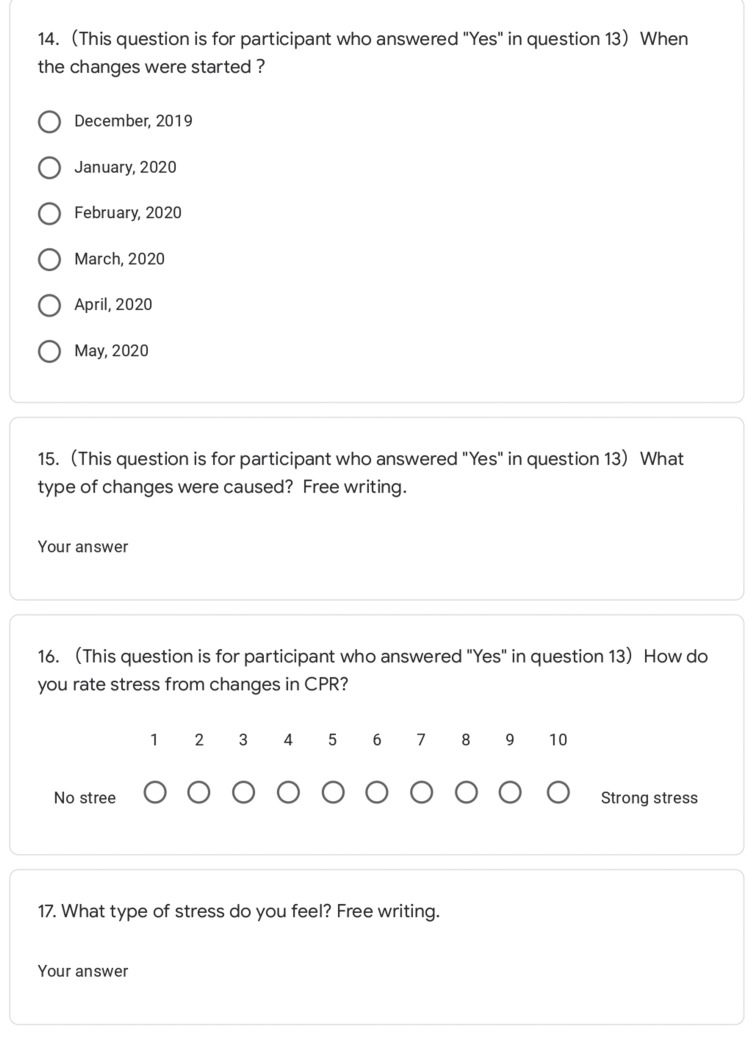
English questionnaire for evaluating changes in the treatment for out-of-hospital cardiac arrest during the initial stage of COVID-19 outbreak, Page 5

**Figure 6:**
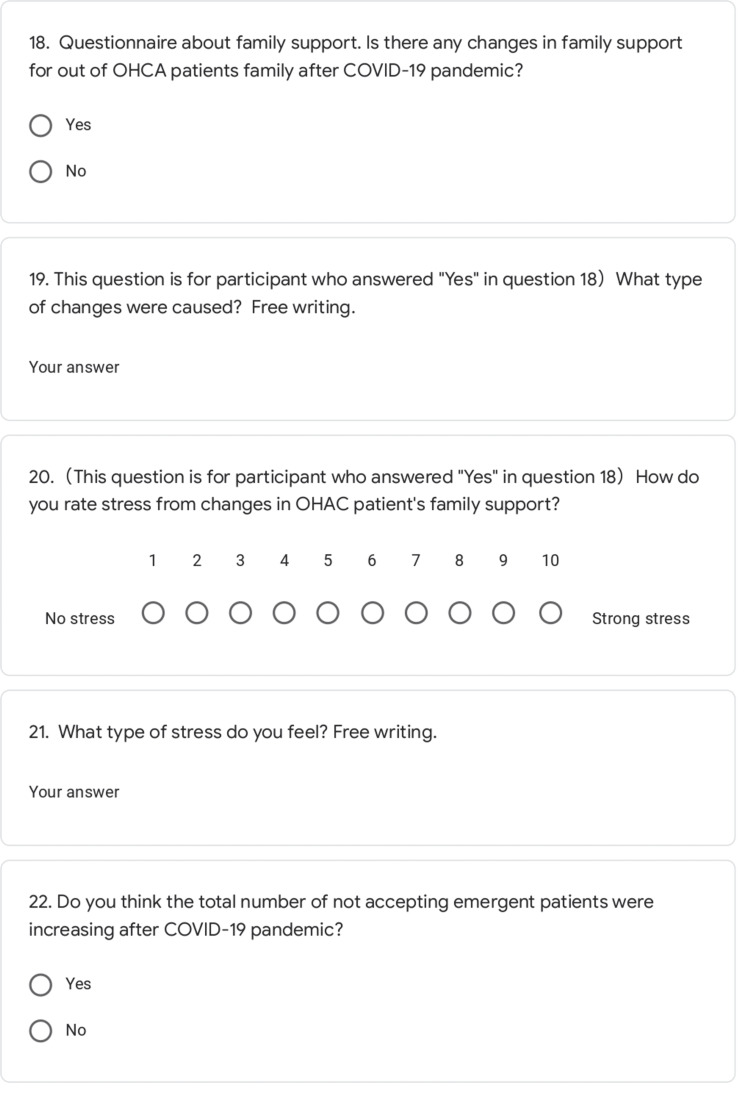
English questionnaire for evaluating changes in the treatment for out-of-hospital cardiac arrest during the initial stage of COVID-19 outbreak, Page 6

**Figure 7:**
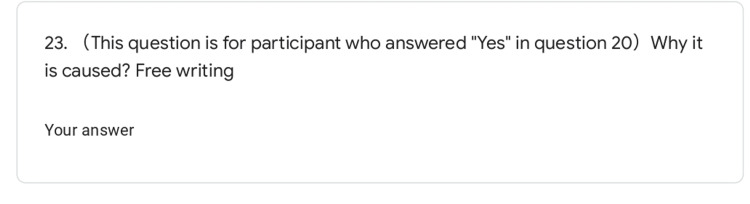
English questionnaire for evaluating changes in the treatment for out-of-hospital cardiac arrest during the initial stage of COVID-19 outbreak, Page 7

**Figure 8:**
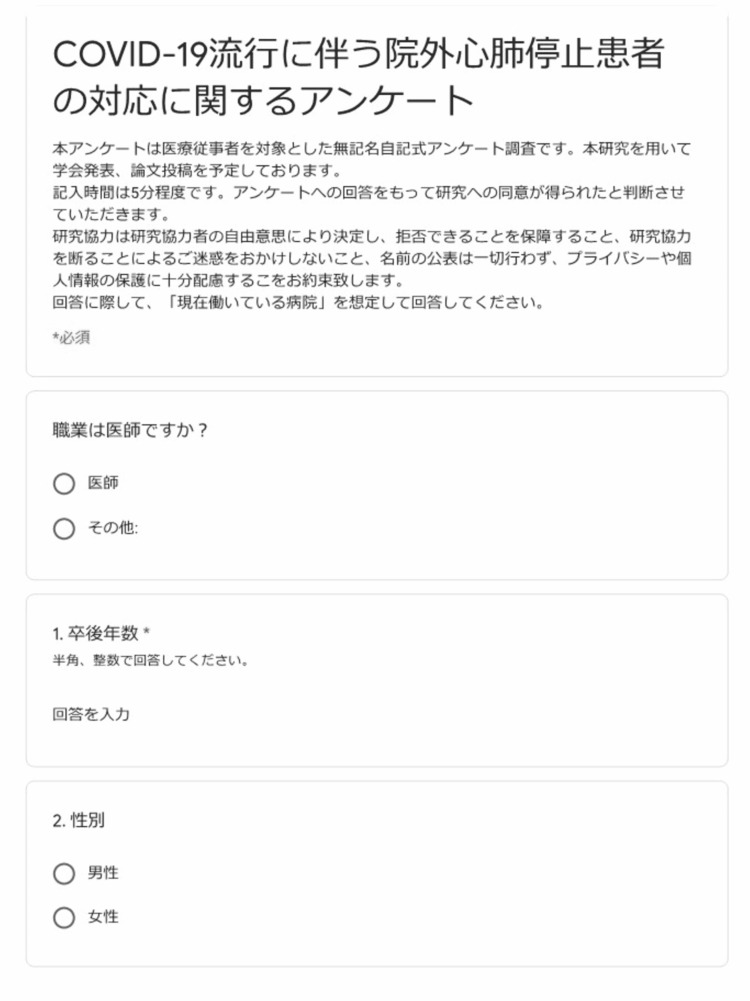
Japanese original questionnaire for evaluating changes in the treatment for out-of-hospital cardiac arrest during the initial stage of COVID-19 outbreak, Page 1

**Figure 9:**
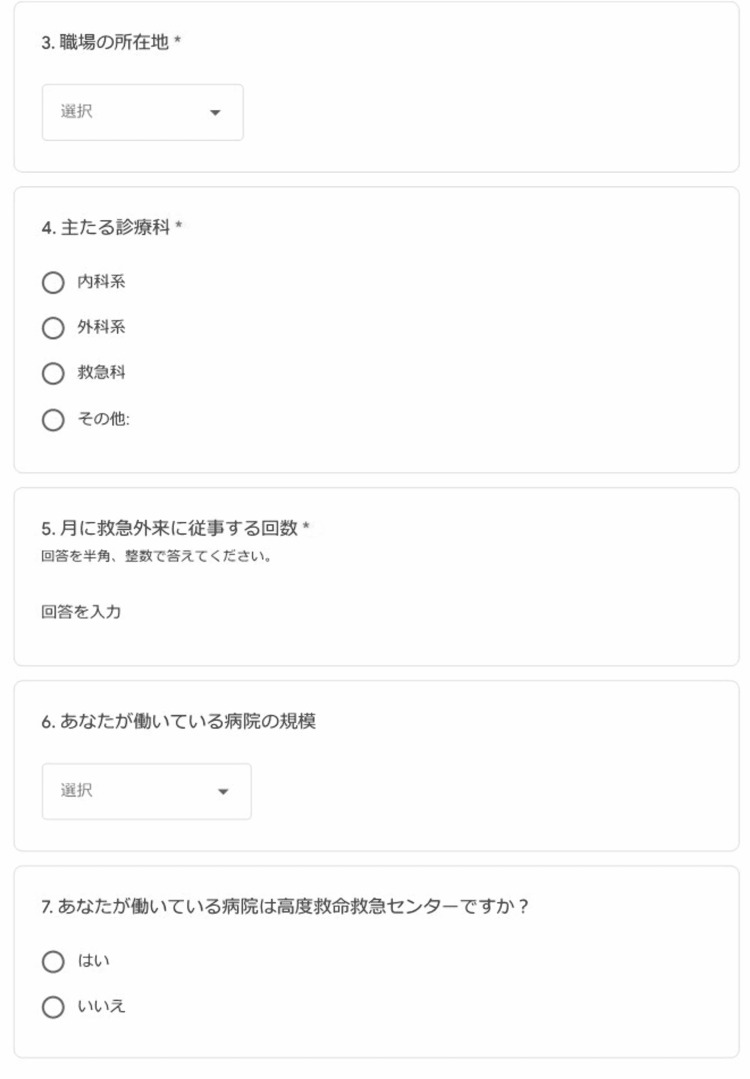
Japanese original questionnaire for evaluating changes in the treatment for out-of-hospital cardiac arrest during the initial stage of COVID-19 outbreak, Page 2

**Figure 10:**
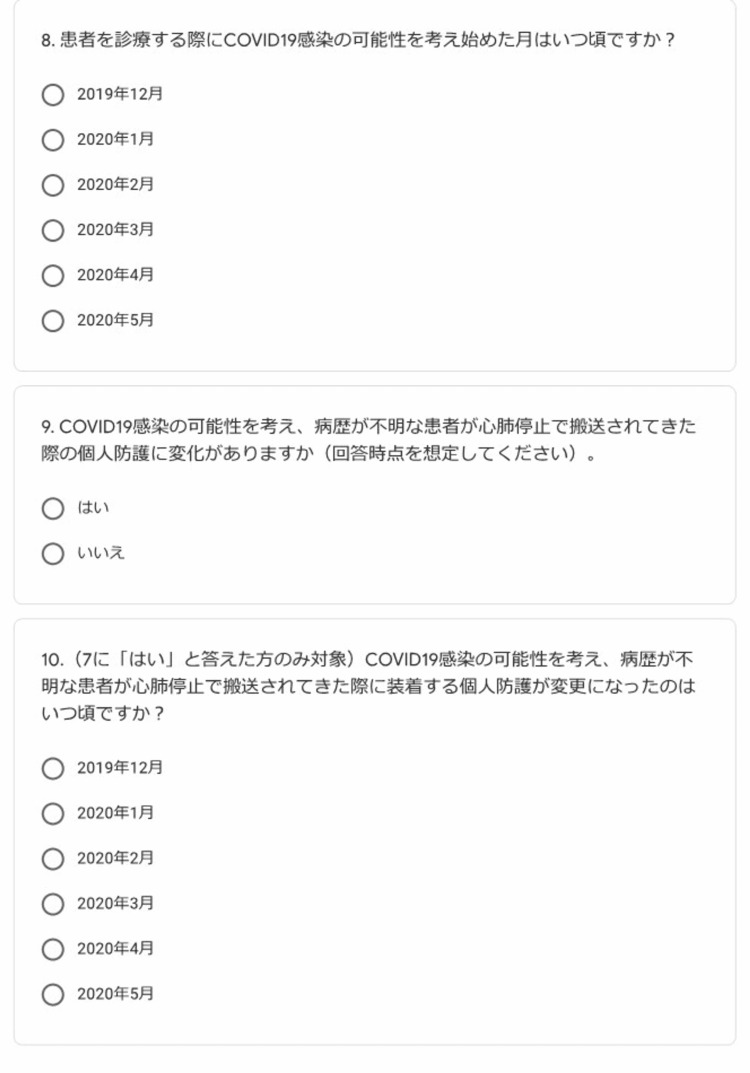
Japanese original questionnaire for evaluating changes in the treatment for out-of-hospital cardiac arrest during the initial stage of COVID-19 outbreak, Page 3

**Figure 11:**
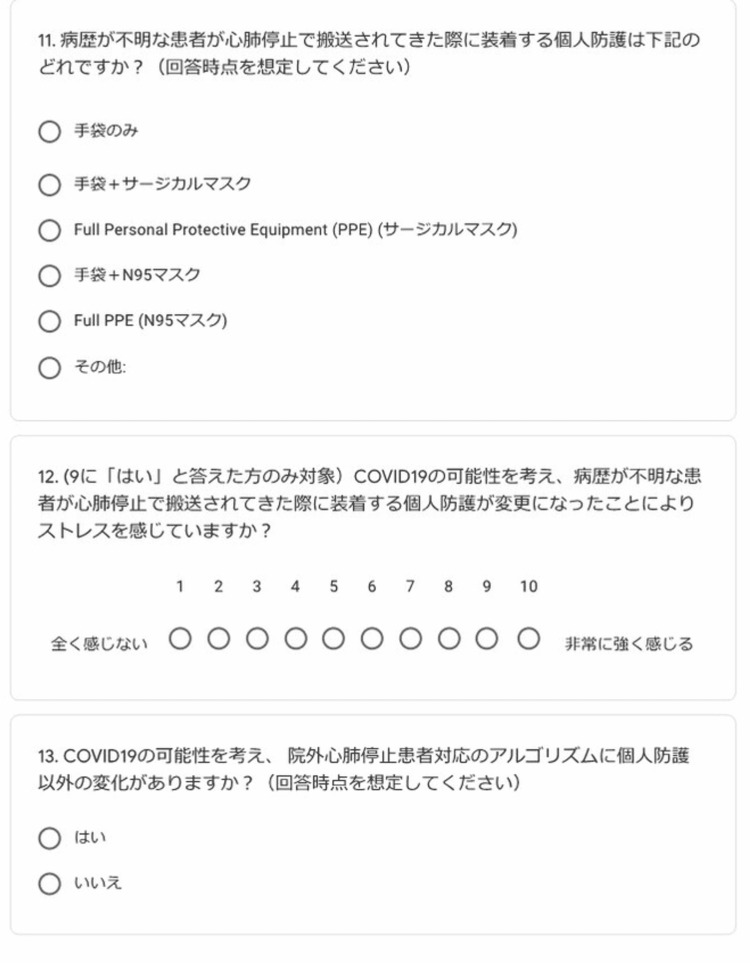
Japanese original questionnaire for evaluating changes in the treatment for out-of-hospital cardiac arrest during the initial stage of COVID-19 outbreak, Page 4

**Figure 12:**
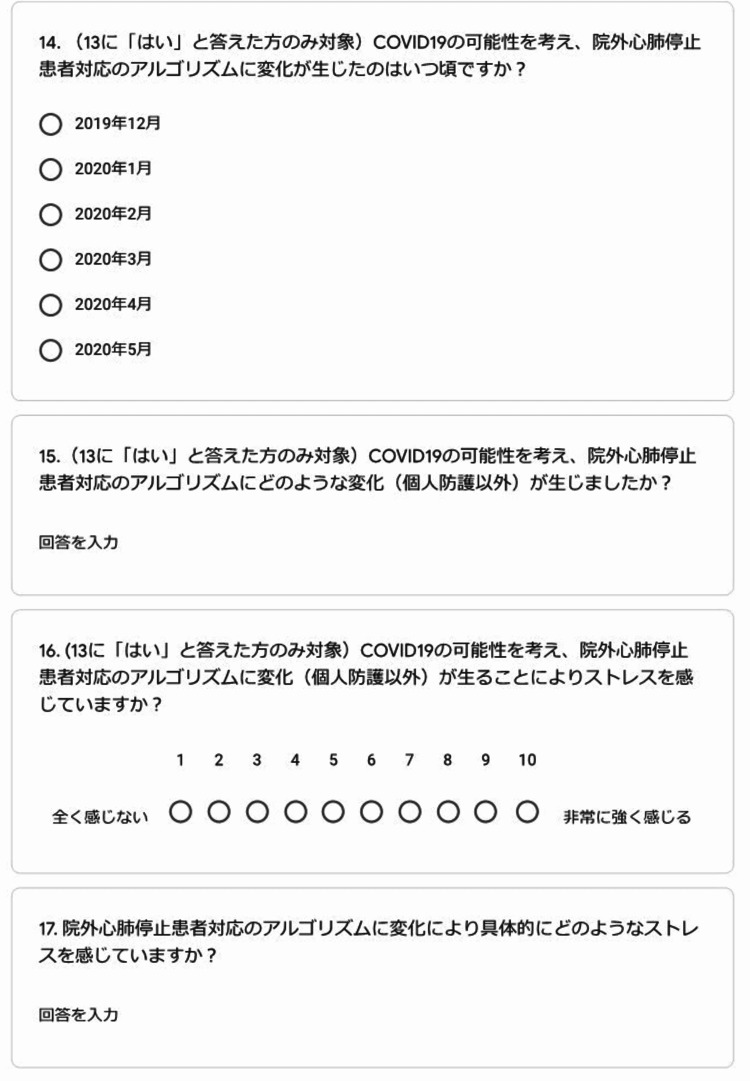
Japanese original questionnaire for evaluating changes in the treatment for out-of-hospital cardiac arrest during the initial stage of COVID-19 outbreak, Page 5

**Figure 13:**
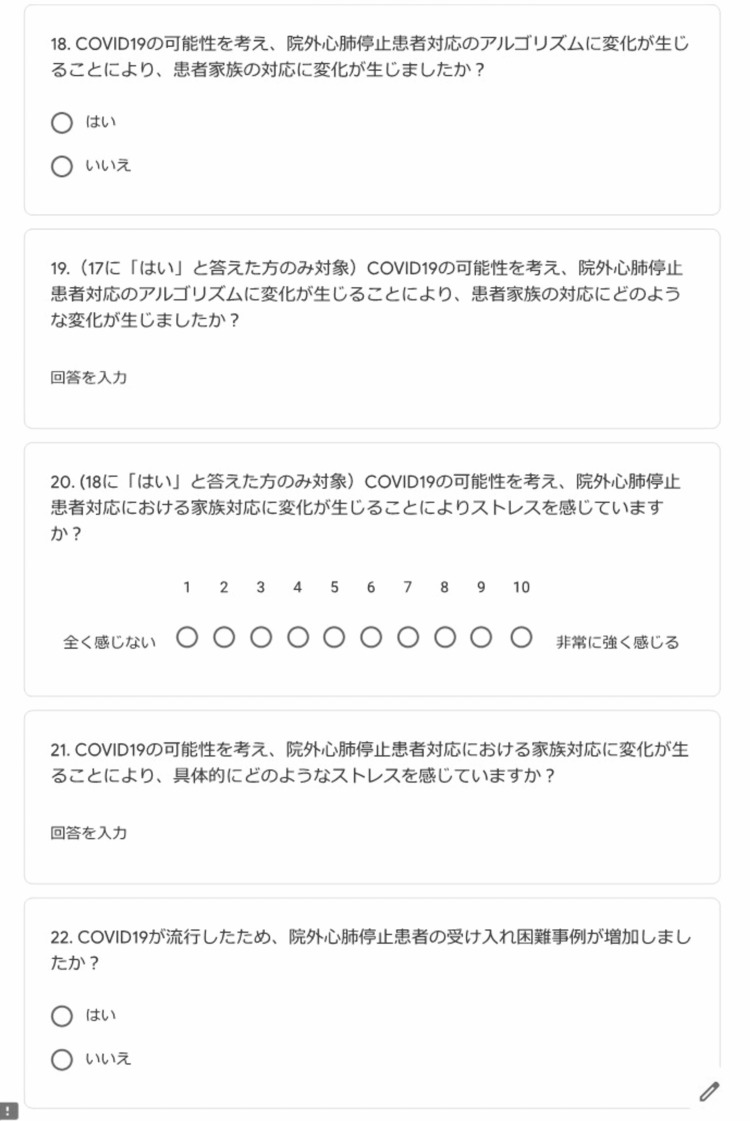
Japanese original questionnaire for evaluating changes in the treatment for out-of-hospital cardiac arrest during the initial stage of COVID-19 outbreak, Page 6

**Figure 14:**
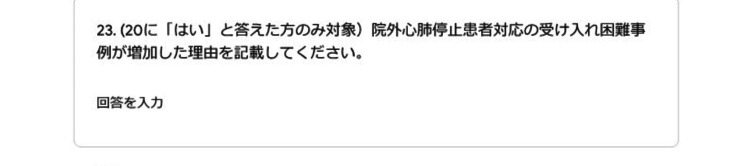
Japanese original questionnaire for evaluating changes in the treatment for out-of-hospital cardiac arrest during the initial stage of COVID-19 outbreak, Page 7
